# Inactivating Effects of Common Laboratory Disinfectants, Fixatives, and Temperatures on the Eggs of Soil Transmitted Helminths

**DOI:** 10.1128/Spectrum.01828-21

**Published:** 2021-12-15

**Authors:** Kristine J. Kines, Mark Fox, MacKevin Ndubuisi, Guilherme G. Verocai, Vitaliano Cama, Richard S. Bradbury

**Affiliations:** a Division of Parasitic Diseases and Malaria, Centers for Disease Control and Prevention, Atlanta, Georgia, USA; b Oak Ridge Associated Universities, Oak Ridge, Tennessee, USA; c Department of Infectious Diseases, College of Veterinary Medicine, University of Georgia, Athens, Georgia, USA; Hebrew University of Jerusalem

**Keywords:** biosafety, STH, helminth, nematode, parasite, inactivation

## Abstract

Soil-transmitted helminths (STH) are important and widespread intestinal pathogens of humans and animals. It is presently unknown which inactivating procedures may be universally effective for safe transport, preservation, and disinfection of STH-contaminated specimens, and this lack of knowledge may expose laboratory staff to higher risk of laboratory-acquired infections (LAI’s). There are limited data on the efficacy of commonly used disinfectants and fecal fixatives for inactivating the eggs of STH. This work tested five disinfectants for surface cleanup, four storage temperature conditions, and six transport/storage fixatives, to inactivate eggs of three species of STH of animal origin (*Ascaris suum* “roundworm,” *Trichuris vulpis* “whipworm” and Ancylostoma caninum “hookworm”) as surrogates for human STH. Among disinfectants, exposure to 10% povidone-iodine for ≥5 min inactivated 100% of the three species tested, while 5 min exposure to 95% ethanol inactivated *T. vulpis* and A. caninum eggs. All of the fixatives tested had inactivation effects on A. caninum hookworm eggs within 24 h of exposure, except potassium dichromate, which required 48 h. 95% ethanol for ≥48 h inactivated eggs from all three STH species. Freezing at ≤−20°C for ≥24 h inactivated eggs of *T. vulpis* and A. caninum, but only freezing at −80°C for ≥24 h inactivated >99% eggs, including *A. suum*. This work provides an evidence base for health and safety guidelines and mitigation strategies for the handling, storage, and disposal of stool samples containing STH eggs in laboratory, health care, childcare, or veterinary settings.

**IMPORTANCE** This study systematically evaluates common laboratory disinfectants and storage conditions for their effectiveness in inactivating the infective stages of soil-transmitted helminths (STH). Animal-infecting proxy species were chosen to represent three major groups of STH that infect humans: roundworms, whipworms, and hookworms. Previously published work in this area typically focuses on a particular inactivation method, either for a single STH species, or on a subset of closely related species. Because prediagnostic fecal specimens must be regarded as potentially infectious with a mix of species, such information may be of limited utility in a working laboratory. We provide a straightforward summary of storage and disinfection methods that can achieve complete inactivation across a range of STH species, which represents a significant advance for clinical, veterinary and research laboratory biosafety.

## INTRODUCTION

The soil-transmitted helminths (STH) of humans (*Ascaris lumbricoides*, Trichuris trichiura, and hookworms of the genera *Ancylostoma* and *Necator*) represent a major cause of morbidity in human populations worldwide. These intestinal worms represent a public health problem in many developing countries (1, 2) and also affect marginalized communities within some industrialized nations (3, 4). STH infections reduce the economic viability of individuals and nations, with over one million years lost to disability (YLD) globally being attributed to these infections. Ascariasis alone causes 604,000 YLDs, trichuriasis 213,000 YLDs and hookworm disease 845,000 YLDs (5). In accordance with the World Health Organization’s commitment to end STH as a public health problem by 2030 (6), there has been increasing activity and effort in the neglected tropical diseases (NTD) community on the diagnosis, surveillance and control of STH infections globally (1). This increased focus of research and public health activity has led to a worldwide increase in laboratory analysis of STH-contaminated samples, highlighting the need for robust protocols to prevent laboratory-associated infections (LAIs). The occurrence of LAIs with both human and zoonotic hookworms and *Ascaris* spp., as well as the risk factors associated with such infections, have previously been reported (7, 8). Despite the risk, there are no effective universal methods for the simultaneous inactivation of eggs from all species of STH. These risks of STH infection from fecal samples extends beyond laboratory personnel to staff in childcare facilities and schools, medical clinics and hospitals, and veterinary clinics and shelters.

For STH laboratory diagnosis, fresh human fecal samples are most often processed using the Kato-Katz, McMaster, FLOTAC and Mini-FLOTAC diagnostic techniques (9, 10). methods which have inherent LAI risks, the most immediate being exposure to infective larvae of hookworms in samples more than three to 5 days old. Samples kept at room temperature for more than 3 weeks may still contain infective-stage eggs of *T. trichiura* (11), while the time to larval development in *Ascaris suum* is 17–22 days after passage (12). Furthermore, bench spills of fresh feces, or egg and larval concentrates from flotation procedures, may leave microscopic immature STH stages on laboratory surfaces or containers which may survive and develop to their infective stages if not effectively inactivated. The risk of LAI may be higher for some laboratories than others (i.e., where submission of fresh stool with high STH parasite loads is routine), and may be substantially reduced by the consistent adoption of universal safety precautions. Nevertheless, it is important to have universal protocols to address the infectious potential of fresh or preserved fecal samples and egg/larvae concentrates stored under various laboratory conditions.

There is little information regarding the optimal disinfectants to use for cleaning up STH spills in the laboratory. There is lack of data about the optimal exposure time for inactivating STH-contaminated samples when using common laboratory fecal fixatives. The remarkable resistance of *A. lumbricoides* eggs to laboratory fixatives and disinfectants has been reported since at least the early 1950s. *Ascaris lumbricoides* eggs are resistant to formalin (13), as well as ethanol, methanol, clorohexidine, sodium hypochlorite and cresol (14). Exposure to 10% povidone-iodine had sustained and effective inactivation of either decorticated *A. suum* eggs (14) or *A. lumbricoides* eggs (15). Naidoo (16) found that inactivation of *A. lumbricoides* eggs in a simulated sludge spill required exposure to 50% sodium hypochlorite for at least 1 h. This concentration and exposure time of sodium hypochlorite exceeds what is practical for routine laboratory or health care surface cleaning protocols. The remarkable resistance of *Ascaris* eggs extends to less commonly employed disinfectants such as activated and deactivated chlorine (17, 18), phenol, cresol, sodium or potassium hydroxide, glutaraldehyde, paraformaldehyde (17), quaternary ammonium compounds (17, 19) and carbolic acid (16). Although exposure to gamma ray irradiation will effectively inactivate all eggs of *A. suum* (20), this approach cannot be recommended because such a measure cannot be practically implemented in most laboratory and health care settings. Freezing at −10°C and −20°C (21) and refrigeration at 5°C for 6 days (22) have been shown to halt the embryonation of *A. suum* eggs compared to room temperature controls.

Inactivation of other human-infecting STH genera has received less attention in the scientific literature. Speare et al. (23) found that 70% ethanol was the most effective disinfectant, killing all Necator americanus larvae after 10 min exposure. Chloroxylenol (Dettol) and 10% formalin were slower in their killing effect, requiring at least 20 min exposure to kill most larvae. Bleach (1%) and 2% glutaraldehyde killed few larvae after 20 min exposure. Exposure to temperatures above 70°C killed 97% of larvae within 5 min (23). Matsusaki (24) conducted comprehensive experiments on the effects of refrigeration on the subsequent viability of *Ancylostoma duodenale* and N. americanus eggs in human feces, and found that the development of both species was somewhat inhibited by cold temperatures (5–7°C) with *A. duodenale* being somewhat more resistant. In 1963, Thitasut found that aqueous iodine solution was efficacious for inactivating the eggs and larvae of various soil transmitted helminths (N. americanus, Ancylostoma caninum, Strongyloides stercoralis, *A. lumbricoides*, Toxocara *canis* and Trichuris muris), though the necessary concentration and duration of exposure varied among the species (15).

The objective of this study was to assess several methods for their potential to inactivate STH eggs in the context of routine laboratory practice: disinfection of surfaces at any stage of the testing, inactivating effects of the fixatives in which fecal samples are submitted, and of the temperature conditions used for storage, both on pre- or postanalytical stages. Findings from this work may provide valuable data to inform laboratories globally when establishing protocols to improve laboratory safety and reduce the risk of STH LAIs.

## RESULTS

**FIG 1:**
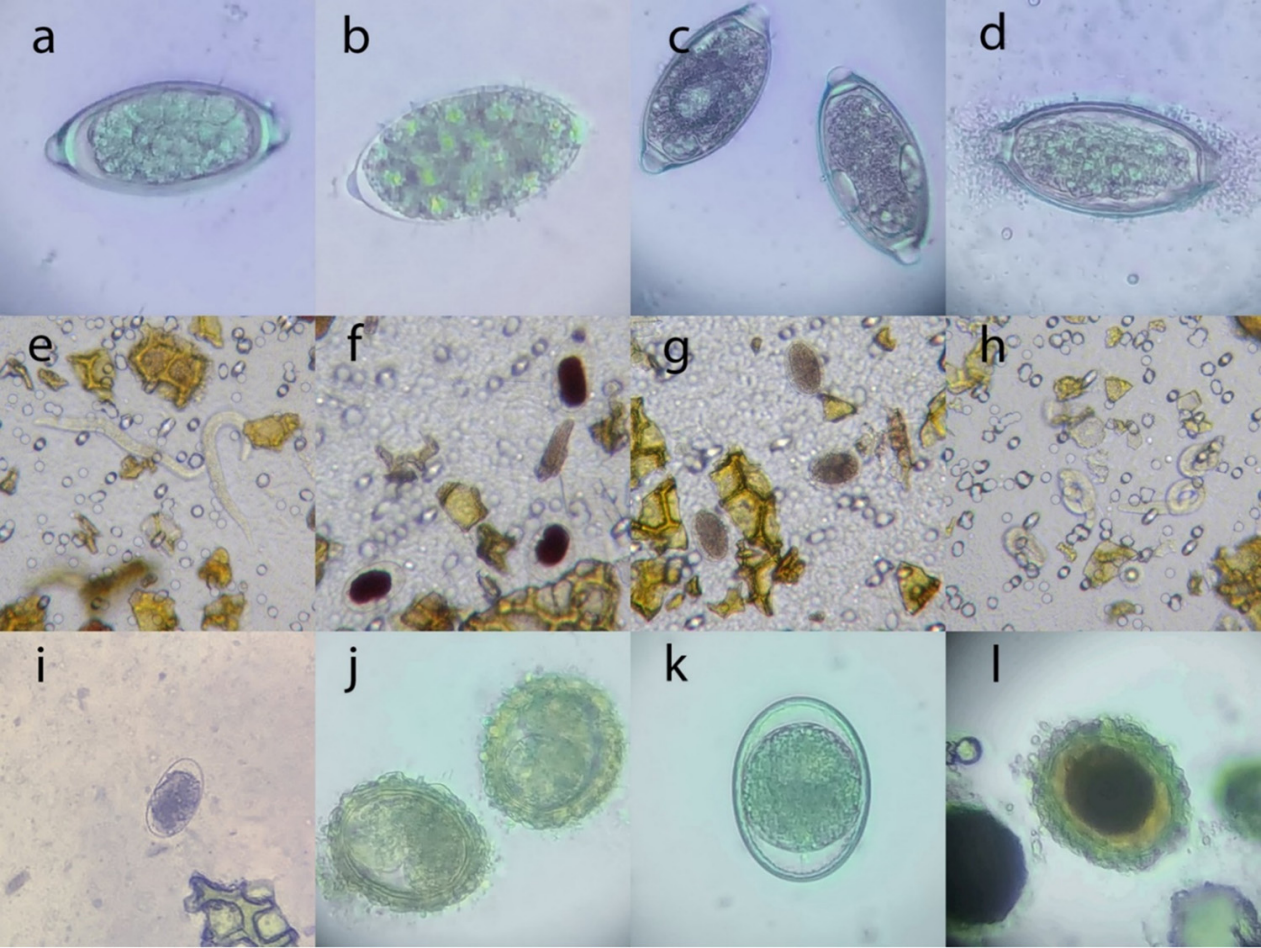
Selected examples of the effects of exposure to common laboratory disinfectants, fixatives, and temperatures on the morphology and development of soil transmitted helminths (STH) eggs. After exposure to listed disinfectants, temperatures or fixative, all STH eggs were allowed to develop for a further 24 h in distilled water before these photographs were taken. (a) *T. vulpis* egg development after 5 min exposure to distilled water (control); (b) *T. vulpis* egg, 5 min exposure to 10% hypochlorite (bleach); (c) two *T. vulpis* eggs, 5 min exposure to 95% ethanol; (d) *T. vulpis* egg, 24 h freezing at −80°C; (e) *A. caninum*, 5 min exposure to distilled water (control); (f) *A. caninum* eggs, 20 min exposure to 10% iodine; (g) *A. caninum* eggs, 20 min exposure to 95% ethanol; (h) *A. caninum*, 20 min exposure to 10% bleach; (i) *A. caninum* egg, 24 h freezing at −80°C; (j) *A. suum* egg, 5 min exposure to water (control); (k) *A. suum* egg, 5 min exposure to 10% bleach; (l) *A. suum* egg, 5 min exposure to 10% iodine.

**FIG 2:**
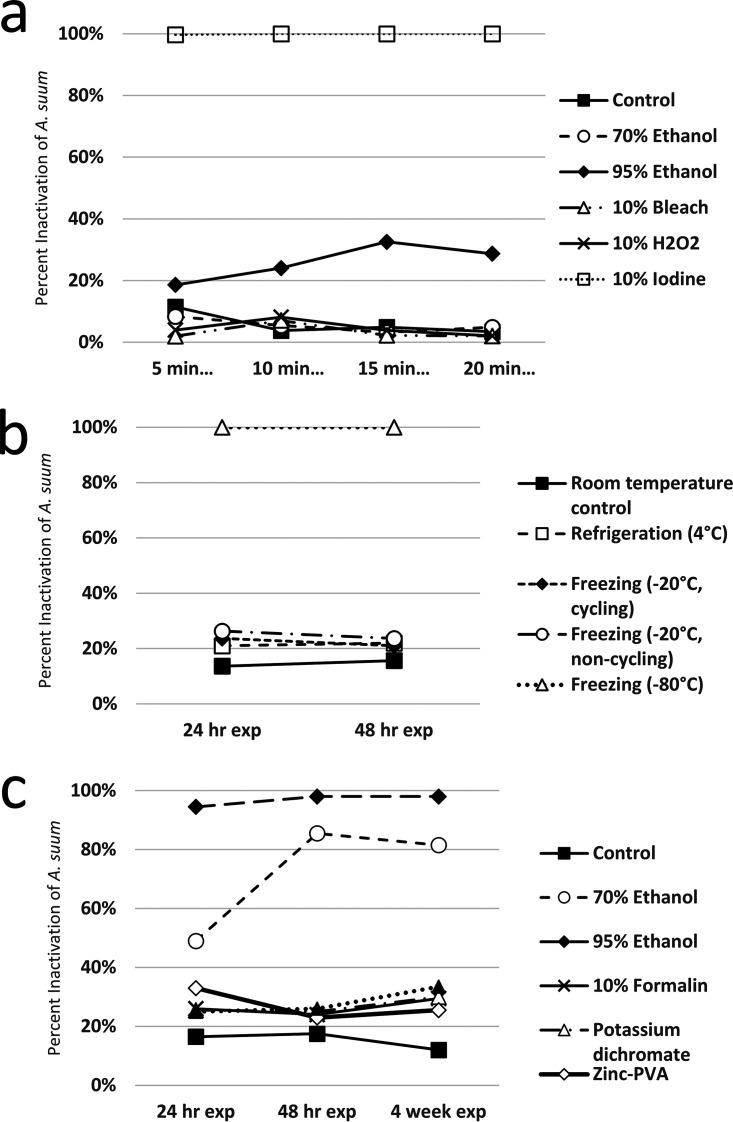
(a) Percent inactivation of *A. suum* eggs following 25 days of culture in distilled water after exposure to several common laboratory disinfectants for 5, 10, 15 and 20 min; (b) inactivation of *A. suum* eggs following 25 days of culture in distilled water after exposure to refrigeration and freezing in various conditions for 24 and 48 h; (c) inactivation of *A. suum* eggs following 25 days of culture in distilled water after exposure to several common laboratory fecal fixatives for 5, 10, 15, and 20 min.

**FIG 3:**
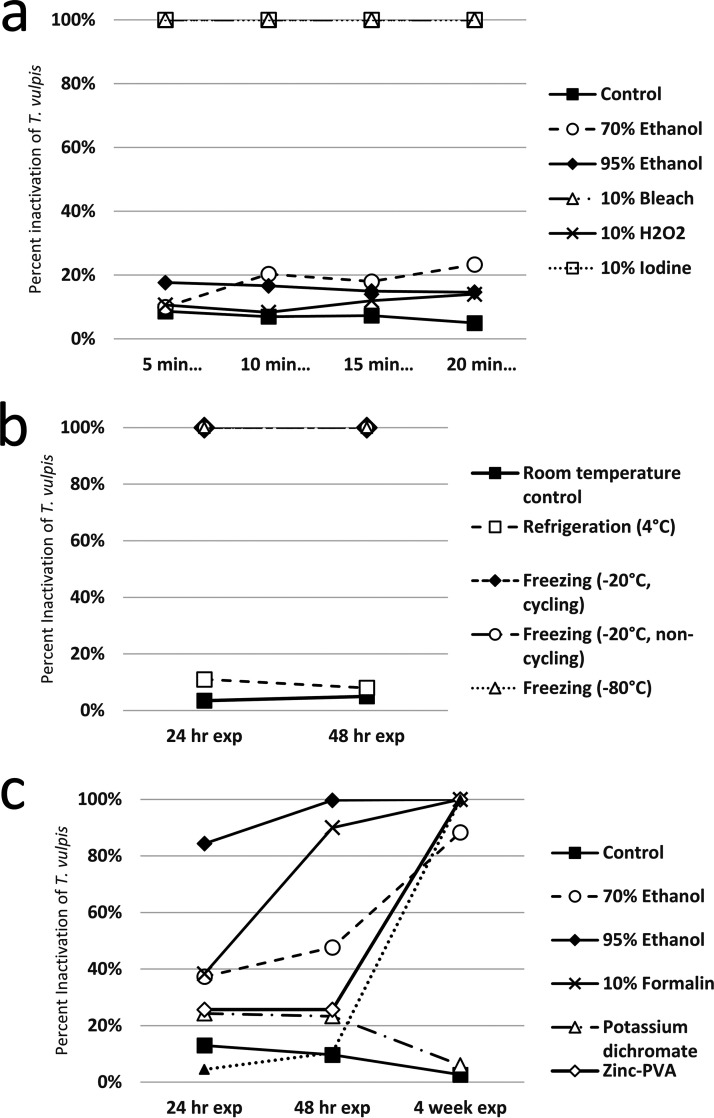
(a) Inactivation of *T. vulpis* eggs following 25 days of culture in distilled water after exposure to several common laboratory disinfectants for 5, 10, 15 and 20 min; (b) inactivation of *T. vulpis* eggs following 25 days of culture in distilled water after exposure to refrigeration and freezing in various conditions for 24 and 48 h; (c) inactivation of *T. vulpis* eggs following 25 days of culture in distilled water after exposure to several common laboratory fecal fixatives for 24 h, 48 h, and 4 weeks (biological duplicate only available for Total-fix values).

A chart summarizing the outcomes of each experiment is presented in Table 1, representative images showing the effects of experimental treatments on egg morphology are presented in Fig. 1, and experiments are individually graphed in Fig. 2, 3, and 4. Table 2 shows the minimum times and concentrations or temperatures required to safely inactivate the eggs or larvae of the STH species we evaluated. Tables S1–3 present the full experimental results, including standard error of the mean (SEM) and statistical significance.

**FIG 4:**
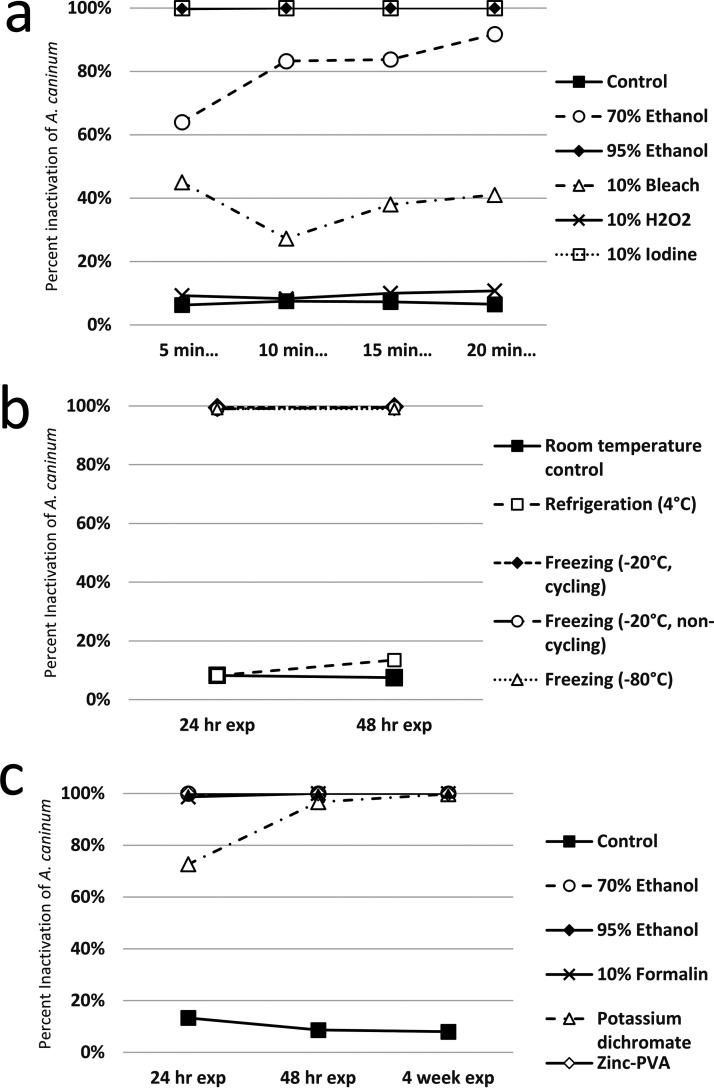
(a) Larval inactivation of A. caninum hookworm eggs (24 h after passage) in distilled water 72 h after exposure of eggs to several common laboratory disinfectants for 5, 10, 15 and 20 min; (b) larval inactivation of A. caninum hookworm eggs (24 h after passage) in distilled water 72 h after exposure of eggs to refrigeration and freezing in various conditions for 24 and 48 h (c) larval inactivation of A. caninum hookworm eggs (24 h after passage) in distilled water 72 h after exposure of eggs to common laboratory fecal fixatives for 24 h, 48 h, and 4 weeks. Biological duplicate only available for 5 min disinfection in 10% bleach values.

**TABLE 1:**
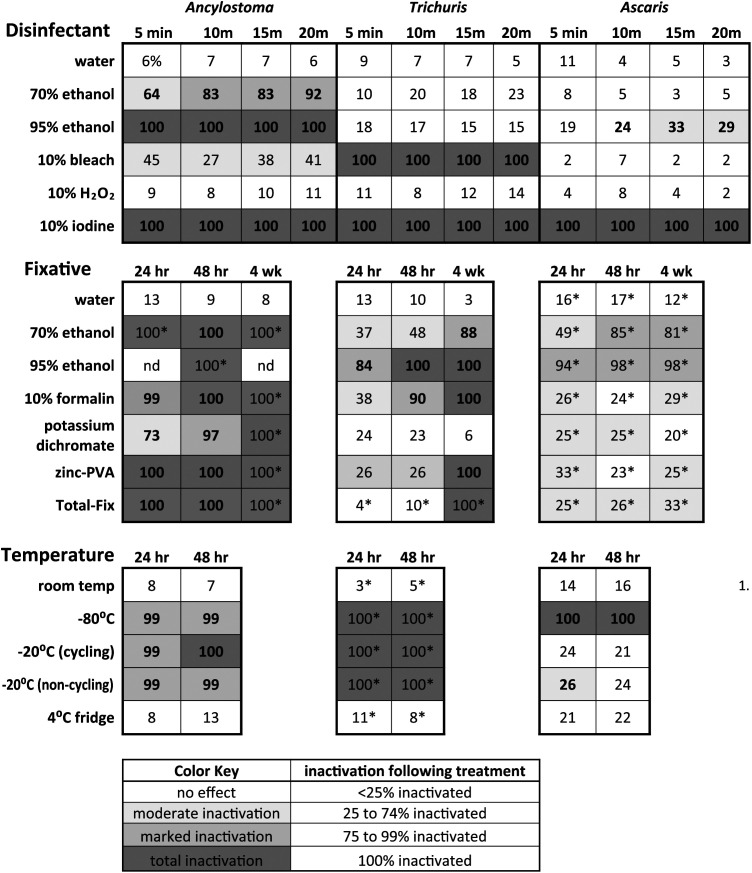
Summary of results

*Indicates experiments that were run in duplicate rather than triplicate, with no *t* test performed.

*^a^*“nd” indicates those data could not be interpreted due to the apparent dissolution of all eggs during treatment. All values are percent inactivation, bolded values indicate significant inactivation versus control (one-tailed paired *t* test, *P* < 0.05).

**TABLE 2:** Minimum times and concentrations/temperatures required to safely inactivate ≥98% of various STH eggs or larvae

Inactivation method	Minimum effective treatment conditions
Eggs of *Ascaris suum*	
Disinfectants	10% Povidone-Iodine for ≥5 minutes
Fixatives	95% ethanol for ≥48 hours
Temperatures	Freezing at −80°C for ≥24 hours
Eggs of *Trichuris vulpis*	
Disinfectants	10% Povidone-Iodine or 95% ethanol for ≥5 minutes
Fixatives	95% ethanol for ≥48 hours; 10% formalin, Zinc PVA for ≥4 wks, or Total-fix for ≥4 wks
Temperatures	Freezing at −20°C (cycling and noncycling), Freezing at −80°C for ≥24 hours
Eggs of Ancylostoma caninum hookworm	
Disinfectants	10% Povidone-Iodine or 95% ethanol for ≥5 minutes
Fixatives	95% ethanol, 70% ethanol, 10% formalin, zinc-PVA for ≥4 wks or Total-fix for ≥24 hours; 5% potassium dichromate for ≥48 hours
Temperatures	Freezing at −20°C (cycling and noncycling), Freezing at −80°C for ≥24 hours

### Disinfectant treatment.

Total inactivation of eggs was achieved for all STH species evaluated after five or more minutes of exposure to 10% iodine, noting that iodine was the only disinfectant to fully inactivate *A. suum* eggs. *Trichuris vulpis* eggs were also completely inactivated by 10% bleach, at every duration of exposure. Ancylostoma caninum eggs were completely inactivated by 95% ethanol at all exposure times evaluated, and marked (≥83%) inactivation was achieved using 70% ethanol for durations of 10 min or more. 10% Hydrogen peroxide was ineffective against all STH eggs tested. There was some variability in effectiveness for other exposure and duration treatments, summarized in Table 1 and the supplementary graphs, but none achieved inactivation results that would justify their use in the event of a laboratory spill.

### Fixative treatment.

No fixative universally and completely inactivated the STH eggs evaluated in our experiments, though 95% ethanol was ≥98% effective for exposures of 48 h or longer. 70% ethanol was completely effective against A. caninum at all durations tested, 88% effective against *T. vulpis* after 4 weeks of storage, and ≥81% effective against *A. suum* for exposures of 48 h or longer. Notably, the ethanol treatments were the only ones to have any meaningful (but not total) inactivating effect on *A. suum*. 10% formalin was ≥99% effective against A. caninum at all exposure times. Formalin was also 90% effective against *T. vulpis* eggs after 48 h of exposure, and totally effective after 4 weeks. Formalin had negligible inactivating effect against *A. suum* eggs. Potassium dichromate was only effective against A. caninum eggs, inactivating 73% after 24 h, 97% after 48 h, and 100% after 4 weeks. Zinc-PVA and Total-Fix were both totally effective against A. caninum eggs at all durations, and totally effective against *T. vulpis* eggs after 4 weeks of exposure.

### Temperature treatment.

Freezing at −80°C for 24 h or more was ≥99%effective at inactivating all the STH species tested, and was the only temperature treatment to inactivate *A. suum* eggs. Freezing at −20°C for 24 h or more was ≥99% effective against A. caninum and *T. vulpis* eggs, and there was no difference between cycling and noncycling freezers. Refrigeration at 4°C had no inactivating effect on any of the STH eggs tested.

### Effects on egg morphology.

While the majority of disinfectants did not alter the morphology of the *A. suum* eggs compared to the distilled water control, some affected morphology (Fig. 1j to l). Microscopy observations showed that bleach removed the corticate membrane from the *A. suum* eggs while iodine was observed concentrated in the central embryo region and in some cases gas bubbles formed within the egg. By temperatures, freezing at −80°C resulted in a shrinking and coalescence of the embryo within the egg but did not distort the external morphology.

Morphological assessment of *T. vulpis* eggs (Fig. 1a to d) showed that those exposed to 95% ethanol had gas bubbles within the embryo, while those exposed to bleach had lost their outer egg wall. Freezing at −20°C and −80°C resulted in a shrinking and coalescence of the embryo within the egg but did not distort the external morphology. This effect was not seen for *T. vulpis* eggs with any of the other fixatives, disinfectants or at refrigeration temperature.

For A. caninum (Fig. 1e to l), exposure to 95% ethanol and 10% iodine completely prevented hatching, with visible iodine concentrating in the embryo of the eggs. Exposure to 70% ethanol resulted in hatching of some larvae, but in some cases, these larvae subsequently died; deterioration of their internal esophageal and intestinal structures was observed. Some eggs exposed to 10% hypochlorite did hatch, but many were nonmotile at 72 h and had curled up. Freezing at −20°C and −80°C and subsequent thawing resulted in inactivation and coalescence of the embryo within the egg but did not distort the outer egg wall (Fig. 1I). Preservation in formalin and the ethanol-based fixatives (70% and 95% ethanol and Total-Fix) resulted in the apparent complete dissolution of some A. caninum eggs.

### Repeatability of assays.

The study was designed to carry out all assays in biological triplicate (each replicate originating from an independent fecal specimen). However, assays to determine the effect of fixatives on *A. suum* eggs were performed only in duplicate, and the analysis of 24 h preservation of *A. suum* eggs in Total-fix was performed only once due to limited sample availability. The effect of Total-fix preservation on *T. vulpis* eggs was also only tested in duplicate due to limited sample availability. Bacterial contamination of hookworm replicates tested in 10% hypochlorite at 5 min necessitated the exclusion of contaminated replicates and although the assay were conducted in triplicate, only duplicate results were available for analysis. This was due to death of hookworm eggs due to overgrowth of contaminants and only two valid replicates were still available for the 4-week fixative exposure. Apparent dissolution of hookworm eggs further complicated the evaluation of several of the fixative experiments. Despite addition of between 200 and 300 eggs per well, two experiments contained no visible eggs on examination at 24 h and 48 h and several others contained fewer than 50 visible eggs or larvae, insufficient to reliably count. The centrifuged pellet of saved liquid from the wash step of one replicate was investigated under the microscope to determine whether eggs had been lost during the washing process; only one egg was found, indicating that there had been no substantial egg loss in the washing step. The bottoms of the culture wells were directly examined using an inverted microscope and no eggs were observed, indicating that eggs had not become affixed to that surface. It is therefore likely that the missing eggs dissolved or deteriorated to the point where they were unable to be detected by light microscopy. If correct, this interpretation would mean the missing eggs had been inactivated, but unfortunately this cannot be proven nor accounted for in our statistical analysis.

### Variability among repeats.

We used SEM to assess variability of the inactivation rates among replicates. Low SEMs were observed among fixatives, disinfectants or temperatures which unequivocally inactivated STH eggs. However, higher SEMs were observed in repeat assays among assays with partial or noneffective inactivation outcomes. assays. This information is fully detailed in Table S3.

## DISCUSSION

For the safe cleanup of spills from laboratory surfaces, disinfection with 10% iodine for a minimum of 5 min was effective across the species tested. To inactivate STH-contaminated fecal specimens for safe transport and processing, preservation in either in 95% ethanol for ≥48 h or freezing at −80°C for ≥24 h inactivated eggs of all tested STH species (including the loss, attributed to dissolution, of hookworm eggs preserved in 95% ethanol). This work represents the first comprehensive study of inactivation methods to include several STH of public health importance and focuses on a practical context in which laboratory workers might risk exposure to STH-infected fecal material: a surface contamination, or when manipulating preserved specimens following storage or transport. These findings are important because eggs of STH are remarkably resistant to many commonly used laboratory disinfectants, fecal fixatives and storage temperatures, and resistance is not consistent across species for most inactivation methods. It is noteworthy that the most effective disinfectants identified in this study (povidone-iodine and, to a lesser extent, ethanol), are also known to be effective against bacteria and viruses which may present a concurrent risk of LAI when handling potentially STH-infected fecal specimens (25).

Although animal STH were used here as surrogates, the data produced are likely to be applicable to the very closely related human STH species and may be directly relevant for laboratory work in a veterinary setting or in the event of a zoonotic infection with these parasites of domestic animals (26, 27–29). Moreover, although A. caninum is a hookworm species of dogs, it may reach patency in some human infections or may only develop to subadult stage in other human infections, leading to eosinophilic enteritis (27).

### The inactivating effects of disinfectant treatment.

Among those evaluated in this study, 10% povidone-iodine was the only disinfectant that universally inactivated all the STH eggs tested, which is consistent with the findings of Thitasut (15). Ancylostoma caninum eggs were also effectively inactivated by 95% ethanol in our study, and *T. vulpis* eggs by 10% bleach, but because of the substantial risk of mixed species infections in STH-areas of endemicity, we cannot recommend these disinfectants for laboratory use in cleaning up potentially STH-contaminated fecal spills. *Ascaris* eggs were resistant to all tested surface disinfection methods except for iodine, which is consistent with previous papers (14, 15, 30) but contrary to the findings of Labare, who reported that povidine-iodine had no effect on corticate *A. lumbricoides* eggs, even at concentrations of up to 100% (23).

### The inactivating effects of chemical fixatives.

We found that 95% ethanol was broadly, though not totally, effective at inactivating the STH eggs tested, when the chemical exposure lasted for 24 h or more. Given their widespread use as fecal fixatives, it is important to note that 70% ethanol was not universally effective at inactivating all STH eggs, and 95% ethanol was most effective against all three STH species when stored for 48 h or more. *Ascaris suum* eggs were quite resistant to every fixative except 70% and 95% ethanol. Hookworms were susceptible to every fixative evaluated in this study, although in several experiments the eggs appear to have dissolved completely and could not be counted. Further study will be required to confirm this inference, but based on our experiments, 10% formalin, ethanol and Total fix may cause significant reduction or complete dissolution of hookworm eggs, and are not recommended as they may affect subsequent egg counts of samples preserved in any of these fixatives, as well as diagnostic sensitivity for ova & parasite examination (28, 29). None of the common laboratory fixatives evaluated in these experiments effectively halted larval development of *T. vulpis* eggs after 24 h exposure, but after 4 weeks of preservation, 70% and 95% ethanol, zinc PVA and 10% formalin all had completely inactivated eggs of *T. vulpis*. The commercial fixative Total-fix caused a marked, nearly complete inactivation after 4 weeks, though some residual embryonation was still observed in Total-fix preserved samples. However, exposure to 5% potassium dichromate for any duration tested did not inactivate *T. vulpis* or *A. suum* eggs, an important consideration for laboratories using this solution to store fecal samples. In the event of a spill of preserved STH-infected stool, which may contain viable eggs, it is possible that the disinfectants found by this study to be effective at inactivating eggs in fresh stool may be used with similar good effect. However, additional experimentation will be required to explicitly demonstrate whether this is the case.

### The inactivating effects of cold storage.

Freezing at −80°C for 24 h or more was universally effective at inactivating the STH species tested. Freezing at −20°C for ≥24 h inactivated *T. vulpis* eggs and A. caninum eggs, but had no significant inactivating effect on *A. suum* eggs. Refrigeration at 4°C had no effect on any of the STH tested, compared to the room temperature control. Therefore, samples shipped or stored frozen may pose little to no risk for *Trichuris* or hookworm infections, whereas samples that have been refrigerated may still become infective. Consistent with the findings of Dziekońska-Rynko, et al. (21), we found that freezing at –20°C only somewhat reduced the percentage of *Ascaris* larval embryonation after subsequent incubation at room temperature. The discrepancy in our refrigeration findings with those of Matsusaki (24), who found that refrigeration inhibited development of *A. duodenale* eggs, may represent species-dependent variation in environmental resistance. It should be noted that freezing resulted in lysis of the cells of the developing larvae (Fig. 1i), a morphological change which should be considered when performing microscopic diagnostic surveillance of hookworms on previously frozen feces.

### Reproducibility and limitations of the assay.

As noted in the results, for some condition/solution/parasite combinations, highly reproducible results were obtained in most of our assay, whereas for others, high variation was seen across experimental replicates. This variability may be influenced by the choice to use biological triplicate rather than parallel triplicate testing of all combinations in this study, with the eggs for each replicate originating from a different animal’s fecal specimen. The multiple sources of eggs and the extended incubation times that provided opportunity for overgrowth of bacteria in some replicates might have contributed to variation in the experimental results. Given these many factors, the reproducible and unequivocal results pertaining to the agents and conditions which caused complete inactivation was a successful outcome.

The *A. suum* assays used washed eggs isolated from pig feces, but in many cases residual bacteria might have remained along the eggs tested. Any bacterial contamination would reduce oxygen availability, which directly impacted embryonation and invalidated the results of some replicates. Antimicrobial agents were not added to combat bacterial growth, as the presence of antibacterials themselves might alter the outcome of experiments in the system. As a result of this obstacle, a large number of *A. suum* replicate assays attempted in this study were excluded from this analysis. Consequently, triplicate results could not be generated for common laboratory fixatives and only biological duplicate results are presented in this paper. Bacterial contamination was not such a problem in assays using eggs or larvae derived from dog feces. Only biological duplicate results were obtained for experiments evaluating the effect of Total-fix preservation on *T. vulpis* eggs. Despite this, the duplicate assays showed relatively low standard errors for a biological system and the data were consistent across time periods assayed, indicating that the duplicate data presented are valid. Similarly, although the 24-h value for Total-fix could not be replicated, the consistent results in the 48 h and 4-week treatments support the validity of this individual result. Ancylostoma caninum eggs proved to be delicate and very susceptible to inactivation or outright destruction by the fixatives tested. Though eggs were lost and uncountable in several 4-week fixative experiments, given the demonstrated efficacy of every treatment after 24 and 48 h of exposure, it is reasonable to infer that exposure at longer times such as 4-weeks would be at least as effective at inactivating hookworm eggs. This is informative and reassuring from the perspective of laboratory safety and should also be kept in mind when selecting appropriate storage and transport fixatives if the maintenance of intact egg morphology is required.

### Morphological effects of preservation.

For STH research, surveillance, or diagnosis that relies on microscopic detection and identification of eggs, it will be important to be mindful of the distorting or destructive effects of the preservation methods tested here. In these instances, it may be necessary to use a preservation method that is less effective at inactivating parasite eggs, but that keeps the required identifying morphological features intact. Extra caution would then be required when manipulating samples because some eggs may still be alive and potentially infectious.

### Conclusions.

STH are endemic in large regions of the world, and coendemicity of *Ascaris*, hookworms and *Trichuris* spp. must be considered when selecting suitable safety precautions for laboratory work. Furthermore, the viability of the samples for their final intended use in the laboratory (e.g., molecular testing, microscopic analysis, etc.) must be considered when choosing the most appropriate fixatives or temperatures for inactivation of STH-contaminated fecal specimens. Data from this study will aid in the determination of appropriate protocols for reducing and minimizing the hazard of STH infections in laboratories and other medical and veterinary settings where occupational exposure may occur. Among the disinfectants evaluated in this study only 10% povidone-iodine was broadly effective at inactivating the STH eggs tested. Among the fixatives, none was universally effective, but 95% ethanol was consistently ≥98% effective for specimens stored more than 48 h (with the caveat that hookworm eggs may dissolve and thereafter be undetectable by microscopy). Freezing for more than 24 h in a –80°C freezer was also broadly effective at inactivating the STH eggs tested. Other inactivating methods evaluated by this study were not completely effective or had inconsistent effects across the STH species tested.

Increased work on STH worldwide has led to the need to determine effective and safe methods of inactivating the eggs of these helminths in the laboratory. The inefficacy of many common laboratory fixatives and disinfectants at inactivating STH eggs, particularly with regard to eggs of *A. suum*, should be noted. This study provides important data which may be used by laboratories in selecting appropriate methods for protecting staff who are manipulating specimens containing STH eggs, and for the effective cleanup of laboratory spills.

## MATERIALS AND METHODS

### Purification and preparation of helminth eggs.

Eggs from the swine STH *A. suum* and the canine STHs A. caninum and *T. vulpis* were used as surrogates for human-infecting parasites in this study. Briefly, diagnostic fecal specimens from farmed pigs and shelter dogs in Georgia were submitted to the Department of Infectious Diseases, College of Veterinary Medicine, University of Georgia and screened for helminth eggs and larvae by direct microscopy on the same day of collection. Leftover fecal samples containing parasites of interest were donated to CDC, where eggs were purified using double-centrifugation flotation in Sheather’s sucrose solution (specific gravity = 1.25), as previously described by Hoggard et al. (31). Eggs were then collected from the flotation supernatant using a 15 ml syringe and passed through a filter membrane of appropriate size: 30 μm for *T. vulpis* and *A. suum* and 15 μm for A. caninum eggs. This membrane was then washed in distilled water to harvest the eggs. *Ascaris suum* eggs were found to be corticated upon the initiation of testing. The mean average count of five individual chambers of a Kova slide (Kova International, Garden Grove, CA) was used to estimate the final egg count of the resulting helminth egg concentrate solution used in experimentation. A minimum initial count of 300 eggs per 100 μl was used for each experiment. All egg concentrate solutions were homogenized by inversion 20 times prior to inoculation into an assay.

### Exposure of STH eggs to disinfectant solutions.

The following disinfectants were tested: Lugol’s iodine diluted to 10% strength (Sigma Chemicals, St. Louis, MO), hydrogen peroxide diluted to 10% strength (Fisher Science Education, Nazareth, PA), commercial bleach (8.25% sodium hypochlorite, Clorox Professional Products Company, Oakland, CA) diluted to 10% of original strength, 70% ethanol, and 95% ethanol (Sigma Chemicals, St. Louis, MO).

For *T. vulpis* and *A. suum* eggs, the following protocol was used: an aliquot of ∼100 μl containing roughly 300 eggs was added to 15 ml conical tubes tube and exposed to 900 μl of each disinfectant or the distilled water negative control at room temperature for five, 10, 15, and 20 min. Twenty minutes was arbitrarily chosen as the maximum contact time for these experiments as longer exposure times were deemed impractical for routine cleanup procedures in a busy laboratory. At the end of the chosen incubation period, material in tubes was washed twice with 10 ml of 1× phosphate-buffered saline (PBS, pH 7.4) to remove the disinfectants, after each wash the tubes were immediately centrifuged at 500 × *g* for 5 min, and the supernatant removed. A final wash with 15 ml of PBS was added to the tube, the pellet was resuspended, and the sample centrifuged again. The final egg pellets were resuspended in 1 ml of distilled water and the total volume of each sample transferred to a well of a 24-well plate and incubated in a fume hood at room temperature (23–27°C) for 25 days.

For A. caninum experiments, the protocol was as above, except that instead of tubes, the eggs were aliquoted into 12 μm pore diameter transwell filter inserts (MilliporeSigma Millicell Culture Plate Inserts, Thermo Fisher Scientific, Waltham, MA), to be placed in 24-well plates with the different disinfectant treatment. This variation was employed to avoid centrifugation, as hookworm eggs are delicate and may be destroyed when centrifuged. After exposure, the transwell cups were promptly placed on a blotting sheet of tissue to draw out any remaining disinfectant and then washed twice with 1× PBS by filling the transwell cup and drawing the PBS across the membrane into a clean tissue by capillary action. Because A. caninum eggs hatch within 1–2 days and larvae are infectious within 5–7 days (26), the transwell cups with hookworm eggs were placed into 24-well plate as above, incubated for 72-h at room-temperature, and counted.

For *Ascaris* and *Trichuris*, the incubation periods postexposure were based on published larval development times of each species: *A. suum* eggs are infectious 18 or more days after passage (16); *Trichuris* eggs are infectious within 15–30 days after passage, with *T. vulpis* eggs developing to an infectious state within 18 days in laboratory culture (32). The incubation protocol consisted of topping up wells with distilled water and stirring daily using a toothpick to ensure adequate oxygen diffusion and avoid desiccation.

Assays were set up in biological triplicate, with replicates originating from independent fecal samples. To assess inactivation, all contents from experimental tubes or transwell membranes were microscopically examined to evaluate STH viability. An observation of ≥100 individual eggs/larvae was required for an assay to be considered valid.

### Exposure of eggs to refrigeration and freezing temperatures.

A 100 μl aliquot of each egg concentrate was added to 900 μl of distilled water in a 24-well plate. Eggs were incubated for 24 and 48 h at three different temperatures: 4°C, −20°C, and −80°C. Both noncycling −20°C freezers and frost-free (referred to here as “cycling”) −20°C freezers were evaluated. A room temperature incubation (temperature maintained between 22 and 24°C) was used as negative control. Following temperature exposures, egg concentrates were incubated in 24-well plates as described for the disinfectant study samples. Assays were performed in biological triplicate, except *T. vulpis* testing which was in duplicate due to limited sample availability.

### Exposure of eggs to frequently used laboratory stool fixatives.

The following laboratory chemicals were tested: 10% formalin (Sigma Chemicals, St. Louis, MO), 5% potassium dichromate (Sigma-Aldrich Co, St. Louis, MO), Total-fix (Medical Chemical Corporation, Torrance, CA), zinc sulfate-polyvinyl alcohol (zinc-PVA; Medical Chemical Corporation, Torrance, CA), 70% ethanol and 95% ethanol (Sigma Chemicals, St. Louis, MO). Inactivation assays were similar to the disinfectant studies, except that the egg concentrates were exposed to each fixative for 24 h, 48 h and 4 weeks at room temperature in 15 ml tubes. For hookworm eggs, the exposure took place in *transw*ell membrane filter cups placed in the wells of a 24-well plate to avoid centrifugation. Egg concentrates were then washed and incubated in the 24-well plates as described for the disinfectant study. Assays were performed in triplicate, except in some cases where limited sample availability forced fewer replicates. Due to apparent loss of hookworm eggs in some treatments, inclusion criteria for the A. caninum fixative experiments differed: replicates were included if living eggs or larvae were observed in the water control, and at least 50 individual eggs or larvae could be counted in four or more fixative treatments.

### Assessment of inactivation by measurement of subsequent viability.

To assess the inactivation of *T. vulpis* and *A. suum* eggs following treatment or incubation, the entire 1 ml volume of each well was mixed, transferred to a 1.8 ml microtube and pulse-spun for 10 s in a mini-centrifuge (max 12.5k rpm). Using a micropipette, 950 μl of supernatant was removed, and the eggs were resuspended in the remaining 50 μl of distilled water. Two 25 μl aliquots of the resuspended eggs were transferred to a microscope slide and eggs and larvae counted using a compound microscope (×100 magnification). For assessment of A. caninum egg viability, eggs were incubated and counted within their transwell filter cup. Assessment of viability was conducted by visually observing the presence of a developed larva within the egg for *A. suum* and *T. vulpis*. For hookworm eggs, the number of rhabditiform larvae hatched, or developed and motile within eggs was used to determine viability. Embryonation rates were expressed as a percentage of all eggs/larvae present, with inactivation expressed as (100% – embryonation%). The efficacy of treatment was also visualized using a scale of four categories based on absolute percentage viability after treatment. These were: no effect (less than 25% inactivation), moderate effect (25–74% inactivation), marked effect (75–99% inactivation) and complete (100%) inactivation.

### Investigation of wash supernatant for loss of hookworm eggs.

Liquid from the initial egg wash step of one replicate of the 95% ethanol preservation experiment was centrifuged at 500 g for 5 min and the deposit screened by direct microscopy for the presence of hookworm eggs, to evaluate whether hookworm eggs were being lost when being transferred as opposed to bursting or becoming optically invisible due to fixative exposure.

### Statistical analyses.

A one-tailed, paired *t* test was used to evaluate each experiment for which we achieved three or more replicates. The one-tailed alternative hypothesis was that a given treatment would have a greater inactivating effect than the control (i.e., a greater proportion of the 100 eggs would be dead/inactivated). For experiments with fewer than three replicates, mean and standard error of the mean (SEM) were noted, but not statistically tested. Mean of individual experiment results and *t* test for comparison of results were calculated using the statistical analyses package of Microsoft Excel in Office 365. Standard error of the mean was calculated in Microsoft Excel in Office 365.
