# Supplementary material for: Inactivating Effects of Common Laboratory Disinfectants, Fixatives, and Temperatures on the Eggs of Soil Transmitted Helminths
Source: Microbiol Spectr. 2021 Dec 15;9(3):e01828-21. doi: 10.1128/Spectrum.01828-21 (PMC8672909; doi:10.1128/Spectrum.01828-21)
Supplement: SUPPLEMENTAL FILE 1 — Supplemental material. Download SPECTRUM01828-21_Supp_1_seq1.pdf, PDF file, 0.1 MB [file spectrum01828-21_supp_1_seq1.pdf]

	Average Percent Larval Inactivation							
**Disinfectant solutions**	5 min.	S.E.M.	10 min.	S.E.M.	15 min.	S.E.M.	20 min.	S.E.M.
	Ascaris suum							
Control	11%	11%	4%	2%	5%	3%	3%	1%
70% Ethanol	8%	7%	5%	2%	3%	3%	5%	1%
95% Ethanol	19%	7%	**24%**	2%	**33%**	3%	**29%**	6%
10% Bleach	2%	1%	7%	5%	2%	2%	2%	2%
10% H_2O_2	4%	2%	8%	5%	4%	3%	2%	2%
10% Iodine	**100%**	0%	**100%**	0%	**100%**	0%	**100%**	0%
	Trichuris vulpis							
Control	9%	2%	7%	2%	7%	2%	5%	2%
70% Ethanol	10%	4%	20%	16%	18%	13%	23%	17%
95% Ethanol	18%	10%	17%	11%	15%	11%	15%	7%
10% Bleach	**100%**	0%	**100%**	0%	**100%**	0%	**100%**	0%
10% H_2O_2	11%	3%	8%	1%	12%	4%	14%	5%
10% Iodine	**100%**	0%	**100%**	0%	**100%**	0%	**100%**	0%
	Ancylostoma caninum							
Control	6%	2%	8%	2%	7%	1%	6%	1%
70% Ethanol	**64%**	10%	**83%**	3%	**84%**	6%	**92%**	5%
95% Ethanol	**100%**	0%	**100%**	0%	**100%**	0%	**100%**	0%
10% Bleach	45%	20%	27%	12%	38%	20%	41%	20%
10% H_2O_2	9%	1%	8%	2%	10%	2%	11%	3%

10% Iodine	**100%**	0%	**100%**	0%	**100%**	0%	**100%**	0%

Supplementary table 1: Average percent inactivation of *A. suum*, *T. vulpis*, and *A. caninum* eggs following exposure in solution at 1:1 dilution with several common laboratory disinfectants for 5, 10, 15 and 20 minutes in biological triplicate (biological duplicate only available for 5 minute 10% bleach disinfection of *A. caninum* eggs). Assays where the standard error of the mean (S.E.M.) of the biological triplicate assays was >10% are highlighted in red. Statistically significant (1-tailed paired t-test, $p<0.05$) inactivation effects are highlighted in bold.

	Average Percent Larval Inactivation			
**Temperature Conditions**	24 hr.	S.E.M.	48 hr.	S.E.M.
	Ascaris suum			
Room temperature control	14%	2%	16%	6%
Refrigeration (4°C)	21%	10%	22%	13%
Freezing (-20°C, cycling)	24%	8%	21%	8%
Freezing (-20°C, non-cycling)	**26%**	1%	24%	4%
Freezing (-80°C)	**100%**	0%	**100%**	0%
	Trichuris vulpis			
Room temperature control	4%	1%	5%	3%
Refrigeration (4°C)	11%	3%	8%	3%
Freezing (-20°C, cycling)	100%	0%	100%	0%
Freezing (-20°C, non-cycling)	100%	0%	100%	0%
Freezing (-80°C)	100%	0%	100%	0%
	Ancylostoma caninum			
Room temperature control	8%	2%	8%	2%
Refrigeration (4°C)	8%	2%	14%	2%
Freezing (-20°C, cycling)	**100%**	1%	**100%**	0%
Freezing (-20°C, non-cycling)	**99%**	1%	**100%**	1%
Freezing (-80°C)	**99%**	1%	**99%**	1%

Supplementary table 2: Average percent inactivation of *A. suum*, *T. vulpis*, and *A. caninum* eggs, following exposure to refrigeration and freezing in various conditions for 24 and 48 hours (in biological triplicate, except *T. vulpis*, which was tested in biological duplicate). Assays where the standard error of the mean (S.E.M.) was >10% are highlighted in red. Statistically significant ($p<0.05$) inactivation effects are highlighted in bold.

	Average Percent Larval Inactivation					
**Fixative solutions**	24 hr.	S.E.M.	48 hr.	S.E.M	4-week	S.E.M.
			Ascaris suum			
Control	17%	4%	18%	3%	12%	5%
70% Ethanol	49%	12%	86%	2%	82%	7%
95% Ethanol	95%	4%	98%	1%	98%	0%
10% Formalin	26%	12%	24%	1%	30%	2%
Potassium dichromate	26%	1%	25%	8%	30%	5%
Zinc-PVA	33%	15%	23%	12%	26%	9%
Total-fix®	25%	7%	26%	4%	34%	6%
			Trichuris vulpis			
Control	13%	10%	10%	7%	3%	2%
70% Ethanol	37%	30%	48%	24%	**88%**	12%
95% Ethanol	**84%**	5%	**100%**	0%	**100%**	0%
10% Formalin	38%	30%	**90%**	5%	**100%**	0%
Potassium dichromate	24%	17%	23%	19%	6%	2%
Zinc-PVA	26%	21%	26%	18%	**100%**	0%
Total-fix®	5%	1%	11%	5%	**100%**	0%
			Ancylostoma caninum			
Control	13%	3%	9%	3%	8%	5%
70% Ethanol	100%	0%	**100%**	0%	100%	0%
95% Ethanol	nd	nd	nd	nd	nd	nd

10% Formalin	**99%**	1%	**100%**	0%	100%	0%
Potassium dichromate	**73%**	22%	**97%**	3%	100%	0%
Zinc-PVA	**100%**	0%	**100%**	0%	100%	0%
Total-fix®	**100%**	0%	**100%**	0%	100%	0%

Supplementary table 3: Average percent inactivation of *A. suum*, *T. vulpis*, and *A. caninum* eggs following exposure in solution at 1:1 dilution with several common laboratory fecal fixatives for 24 hours, 48 hours and four weeks (in biological triplicate except for *T. vulpis* in TotalFix®, all treatments of *A. suum*, and for *A. caninum* at 4 weeks). Assays where the standard error of the mean (S.E.M.) of the biological triplicate assays (biological duplicate only available for all *A. suum* data, and *A. caninum* at 4 weeks) was >10% are highlighted in red. Statistically significant (1-tailed, paired t-test, $p<0.05$) inactivation effects are highlighted in bold.

nd: no data due to apparent dissolution of eggs in fixative
